# Two‐Dimensional Ketone‐Driven Metal–Organic Coordination on Cu(111)

**DOI:** 10.1002/chem.201600368

**Published:** 2016-04-13

**Authors:** Ada Della Pia, Massimo Riello, James Lawrence, Daphne Stassen, Tim S. Jones, Davide Bonifazi, Alessandro De Vita, Giovanni Costantini

**Affiliations:** ^1^ Department of ChemistryUniversity of WarwickGibbet Hill RoadCoventryCV4 7ALUK; ^2^ Department of PhysicsKing's College LondonStrandLondonWC2R 2LSUK; ^3^ Namur Research College (NARC) and Department of ChemistryUniversity of Namur (UNamur)5000Belgium; ^4^ School of ChemistryCardiff UniversityPark PlaceCF10 3ATCardiffUK

**Keywords:** nanostructures, density functional theory, scanning tunneling microscopy, self-assembly, surface chemistry

## Abstract

Two‐dimensional metal–organic nanostructures based on the binding of ketone groups and metal atoms were fabricated by depositing pyrene‐4,5,9,10‐tetraone (PTO) molecules on a Cu(111) surface. The strongly electronegative ketone moieties bind to either copper adatoms from the substrate or codeposited iron atoms. In the former case, scanning tunnelling microscopy images reveal the development of an extended metal–organic supramolecular structure. Each copper adatom coordinates to two ketone ligands of two neighbouring PTO molecules, forming chains that are linked together into large islands through secondary van der Waals interactions. Deposition of iron atoms leads to a transformation of this assembly resulting from the substitution of the metal centres. Density functional theory calculations reveal that the driving force for the metal substitution is primarily determined by the strength of the ketone–metal bond, which is higher for Fe than for Cu. This second class of nanostructures displays a structural dependence on the rate of iron deposition.

## Introduction

**Figure 1:**
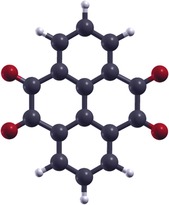
Ball and stick model for pyrene‐4,5,9,10‐tetraone (PTO): dark grey atoms correspond to carbon, red to oxygen and white to hydrogen.

Surface‐based two‐dimensional (2D) metal–organic nanostructures (MOS) are planar and highly ordered networks formed by the coordination of metal centres to tailored functional groups of organic ligands. The incorporated metal atoms confer electronic,^1^ magnetic1b, 2 and catalytic^3^ properties to the network, thereby motivating extensive studies on this class of MOS driven by their potential applications in surface porous material engineering,^4^ catalysis,^5^ and information storage.^6^ The coordination bonding involves charge transfer between the metals and the organic ligands. Determining the oxidation states of the metal centres is relatively straightforward in three‐dimensional (3D) metal–organic frameworks, but more problematic for their 2D counterparts. This is particularly true for polarisable, typically metallic, substrates that can effectively screen charged adsorbates,^7^ and may also contribute to the charge transfer.^1^, ^8^ 2D‐MOS can be built under ultrahigh vacuum conditions by codepositing molecules and alkali,^8^, ^9^ transition,^10^ rare earth^11^ and/or post‐transition metal atoms.^12^ However, such 2D arrangements are also often formed by the incorporation of ‘free’ substrate atoms that are thermally released from step edges and kink sites of metallic surfaces (known as adatoms)^8^, ^13^ or atoms pulled out of the substrate during the molecular binding process.^14^ The geometric arrangement of the resulting assemblies depends on the symmetry and reconstruction of the surface,10a, 15 the choice of metal centres,10a, 16 and the nature and position of the relevant molecular functional groups^12^, 16b that bind to the metal centres and define the nodes of the resulting network. Several molecular moieties, often attached to aromatic rings, have previously been used to fabricate 2D MOS, including cyano/carbonitrile,1b, 17 thiolate13a and carboxylate groups,^18^ poly‐*N*‐heterocyclic compounds,13b, 19 pyridyl groups,10d, 16b, 20 and anhydrides.^21^ Ketone moieties are known to bind to metallic centres and are particularly interesting, owing to their high electron affinity and consequent potential usefulness in organic optoelectronic devices.^22^ Surprisingly, ketone groups have not been extensively used in this context with, to our knowledge, only one very recent example of their application in surface 2D MOS.^23^ In this respect, pyrene‐4,5,9,10‐tetraone (PTO)^24^ (Figure 1) is an ideal molecular candidate for 2D MOS formation, since its equatorial bidentate ketone moieties provide the oxidative potential necessary to stabilise defined oxidation states of the coordination metals. Moreover, the particular arrangement of the two groups on each side of the molecule is expected to control the geometry of the MOS and drive the formation of linear supramolecular assemblies. Herein we present a combined experimental and theoretical study of surface MOS based on the bonding of ketone groups to two different metal centres: Cu adatoms from the Cu(111) substrate and codeposited Fe atoms.

## Results

**Figure 2:**
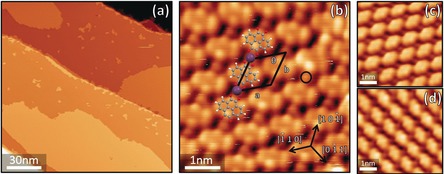
a) STM image of the PTO−Cu islands formed on Cu(111) after annealing to 400 K. The larger islands result from ripening while the smaller ones are most probably located around surface defects or contaminants. b) Close‐up image of the PTO−Cu assembly. A PTO−Cu chain is illustrated within the island with a ball and stick model (Cu atoms are coloured in purple). Unit cell: ***a***=9.6±0.3 Å, ***b***=10.3±0.4 Å, *θ*=54±1°. c, d) The two other orientations of the PTO−Cu chains that were observed on the surface.

PTO was deposited via molecular beam epitaxy on a Cu(111) surface at room temperature under ultra‐high vacuum conditions at sub‐monolayer coverage. Details of the experimental procedure are reported in the Supporting Information. PTO molecules self‐assembled into small islands, evenly spread over the Cu(111) terraces (see the Supporting Information, Figure SI.1). Annealing up to 450 K led to an increase in the size of the molecular islands with a corresponding decrease in their number, without changes to their internal structure, as illustrated by the scanning tunnelling microscope (STM) image (Figure 2 a). Each island consisted of molecular rows aligned along a high‐symmetry surface direction of the Cu(111) substrate (Figure 2 b–d). Along these rows, dimmer circular protrusions between adjacent molecules are clearly evident (circled in black in Figure 2 b). The inter‐row distance between these features is 10.3±0.4 Å, corresponding to four substrate lattice spacings. We identify these protrusions as Cu adatoms bound by the strongly electron‐accepting ketone groups and incorporated in metal–organic rows, composed of alternating PTO molecules and Cu adatoms. The ketone groups are expected to capture the free copper adatoms that are available at room temperature, similar to what was previously observed for other strong electron‐acceptor molecules.^8^, 13b,13c, 25 The metal–organic rows display a secondary organisation level as they are linked together to form extended islands, most probably stabilised by weaker van der Waals interactions. The unit cell for the final supramolecular structure is rhombic, with edge lengths *a*=(9.6±0.3) Å and *b*=(10.3±0.4) Å, and internal angle *θ*=(54±1)°. This geometry was found in the majority of the observed islands, although a few smaller islands with different molecular packing were occasionally observed to grow from step edges (see the Supporting Information).

**Figure 3:**
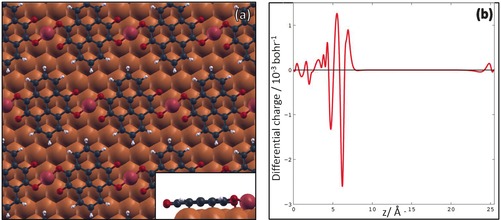
a) DFT‐calculated metal–organic structure formed by PTO molecules and Cu adatoms (coloured in purple) on Cu(111). The inset (side view) shows the flat adsorption conformation of PTO molecules in this assembly. b) Plot of the charge rearrangement at the metal–organic interface, Δ*ρ*(**r**), integrated over the *x*–*y* plane within the unit cell, versus the *z* direction perpendicular to the surface. The 0 of the *z* axis corresponds to the bottom (third) bulk copper layer, that is, the molecule is located at *z*≈6 Å. The two negative peaks correspond to the electron charge transferred from the substrate and from the adatom to the molecule, whereas the positive peaks correspond to the charge accumulated at the molecular core.

We carried out density functional theory (DFT) geometry optimisation for the metal–organic structure revealed by the STM images. The periodically repeated unit cell used in our calculations consists of a four‐layered Cu slab, with an overlayer comprising one PTO molecule and one Cu adatom placed in the three‐fold hollow site of the Cu(111) surface, consistent with what is usually observed for metal atoms on (111) surfaces.^26^ The parameters of our unit cell were *a*=10.21 Å, *b*=10.21 Å, *θ*=60.0°, in good agreement with the experimentally determined values. The calculated minimum energy structure (Figure 3 a) closely matches our experimental observations. Interestingly, the Cu–carbonyl interactions are not equivalent, as only one of the terminal oxygens located at each side of PTO molecules is close enough to directly bind to the adatom (O−Cu bond length: 1.86 Å vs. ca. 2.27 Å for the unbound oxygen). The asymmetric chelation pattern is reflected by the rotation of the molecules, whose long axis is not perpendicular to the direction of the MO rows, but tilted by approximately 5° (this is evident in both the STM rendering and the DFT model; see the Supporting Information, Figure SI.4). The calculated equilibrium bond length is close to the values reported for 3D copper carbonyl^27^ and solution‐phase inorganic complexes.^28^

The simulated STM image (see the Supporting Information, Figure SI.5) also closely agrees with the experimental data at negative bias voltages, that is, for occupied electronic states. PTO molecules are imaged as elongated protrusions, with a shape reflecting the spatial distribution of the lowest unoccupied molecular orbital (LUMO) of a neutral PTO molecule in gas phase, while the Cu adatoms are imaged as bright spots. The calculations hence confirm the assumption that the ketone groups are responsible for the bonding to the Cu adatoms. The resemblance with the spatial distribution of the LUMO at negative voltage (i.e., in occupied states STM imaging) also suggests that the molecules become negatively charged on this substrate. DFT calculations further reveal that the MO coordination is characterised by a significant electron migration from the metal to the organic scaffold, originating both from the adatom and the surface. The calculated charge transfer to the molecule is 2.61 *e* (from the Bader topological analysis of the electron density^29^), resulting in the complete filling of PTO's LUMO (we note that the small molecular adsorption height, ca. 2.3 Å, makes a precise definition of the Bader atomic volumes difficult and might cause an overestimation of the charge transfer in absolute terms). More information on the charge migration can be obtained by calculating the charge rearrangement at the metal–organic interface, Δ*ρ*(**r**), evaluated by subtracting the electron densities of the isolated subsystems (molecule and substrate) from that of the combined system:

$$\Delta \rho \left(𝐫\right)=\rho {}_{\mathrm{int}}\left(𝐫\right)-\left[\rho {}_{\mathrm{sub}}\left(𝐫\right)+\rho {}_{\mathrm{mol}}\left(𝐫\right)\right]$$

where *ρ*_int_(**r**) is the fully interacting electron density and *ρ*_sub_(**r**) and *ρ*_mol_(**r**) are the substrate and molecule densities, respectively, calculated in the gas phase while kept in the adsorbed geometry. By integrating Δ*ρ* over the *x*–*y* coordinates, it is possible to draw a more detailed picture of the charge rearrangement process along *z*. In particular, the two negative peaks in the curve shown in Figure 3 b are associated with the electron charge transferred from the substrate (leftmost negative peak) and from the adatom (rightmost) to the molecule, whereas the two main positive peaks account for the charge accumulated on the symmetric π lobes associated with the aromatic core.

**Figure 4:**
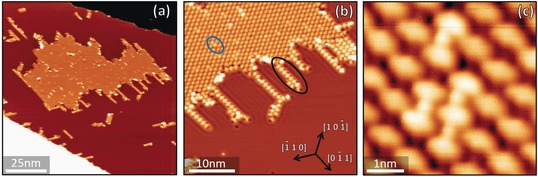
a) Large‐scale STM image of the supramolecular arrangements formed after depositing small amounts of Fe (few percent of a monolayer) at a low rate (see the Supporting Information) onto pre‐existing PTO−Cu assemblies. b) Examples of substitution of Cu for Fe inside PTO−Cu islands (circled in blue) and of PTO−Fe chains developed at the periphery of PTO−Cu islands (circled in black). c) Close‐up of substitutions of Cu for Fe within a PTO−Cu island. Both the Fe atoms and the PTO molecules bound to them appear brighter in the STM images.

Depositing a few percent of a monolayer of Fe atoms at room temperature over the previously formed Cu−PTO assemblies produces the structures shown in Figure 4. Short stripes of alternating PTO and small circular bright protrusions—which we identify as PTO−Fe chains—develop around the boundaries of the PTO−Cu islands (black oval in Figure 4 b), at the edges of the substrate steps, and near small Fe clusters. Bright protrusions can also be observed within the mostly unchanged PTO−Cu islands (blue oval in Figure 4 b and Figure 4 c), which we attribute to cases where individual Cu adatoms have been substituted by Fe adatoms.

**Figure 5:**
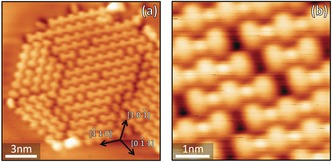
a) Island composed of short PTO−Fe chains developed after annealing the sample depicted in Figure 4 to 450 K. b) Close‐up of a PTO−Fe island.

Annealing to 450 K leads to the appearance of islands made purely of PTO−Fe chains (Figure 5 and Figure SI.6 in the Supporting Information), characterised by a 9.7±0.6 Å average distance between Fe atoms. Within these islands, domains of PTO−Fe chains of varying lengths (average chain length of ca. 4 PTO molecules) are oriented along the substrate high‐symmetry directions and hence rotated by 120° with respect to each other (multi‐domain islands) and separated by grain boundary defects. The STM topographies thus suggest that, upon thermal annealing, PTO molecules are released from the PTO−Cu network and bind with free Fe atoms. This indicates that coordination of PTO to Fe is strongly favoured over that to Cu. Repeating the Fe deposition and 450 K annealing procedure at a higher Fe coverage results in a lower density of multi‐domain islands and the appearance of ordered mono‐domains of PTO−Fe (see the Supporting Information, Figure SI.7).

**Figure 6:**
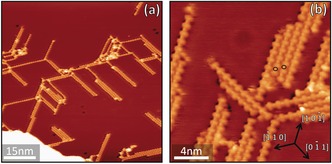
a) PTO−Fe filament structures resulting from depositing an amount of Fe just above a 1:1 ratio with PTO at a high rate (see the Supporting Information) onto pre‐existing PTO−Cu assemblies. b) Close‐up image of several PTO−Fe filaments. Two Fe atoms are circled in black.

In a further experiment, we deposited Fe atoms on top of existing PTO−Cu structures but this time with a (ca. 3 times) higher rate and a (ca. 6 times) higher Fe/PTO ratio, while still holding the sample at 300 K. Surprisingly, the results were quite different from the previous, as no PTO−Cu islands were formed, the surface being instead entirely covered by PTO−Fe stripes. These filaments have an average length of approximately 8 PTO molecules (Figure 6 a), a distance between subsequent Fe atoms of 9.8±0.6 Å—equal to that measured for the lower Fe deposition rate—and a similar alignment with the substrate crystallographic orientations (Figure 6 b). Annealing up to 550 K had no effect on these structures, even if this temperature was significantly higher than that used for the lower deposition rate.

**Figure 7:**
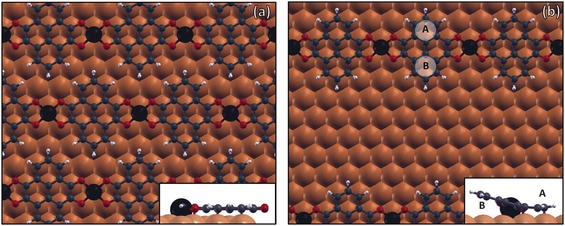
DFT calculations of the two types of PTO−Fe assemblies: a) full PTO−Fe monolayer and b) sparse PTO−Fe filaments. Fe atoms are shown in dark blue. The insets show the side view of the adsorption geometries in the two structures and are taken along the direction of the Fe−PTO chains in (a) and perpendicular to it in (b).

We performed DFT calculations to investigate the structural and electronic properties of the two types of PTO−Fe assemblies, the compact islands and the sparse filaments. We used a 4×4 surface supercell to simulate a full PTO−Fe monolayer, with Fe atoms placed in the three‐fold hollow site on the Cu(111) surface (Figure 7 a). The unit cell for an isolated PTO−Fe stripe (Figure 7 b) consists instead of a four‐layer Cu slab, equivalent to a 8×4 supercell. The resulting minimum energy configuration for the full PTO−Fe monolayer agrees with the experimentally measured unit cell of the compact PTO−Fe multi‐domain islands formed at a low Fe deposition rate (*b*_1_=9.9±0.4 Å, *b*_2_=9.7±0.6 Å, *θ*=59±5°). This supramolecular framework is isostructural to what we found for the PTO−Cu monolayer, with both metal atoms (Fe and Cu) being located in the same three‐fold hollow positions. The calculated Fe‐Fe distance along the bonding axis is 10.21 Å. At odds with the PTO−Cu coordination, all Fe‐carbonyl interactions are equivalent, so that the four carbonyl termini sandwiching the Fe atom are arranged in a square geometry (the O‐Fe bond length is approximately 1.92 Å, close to values reported in other MOS2b, 23, 30 and 3D Fe‐carboxylates metal–organic frameworks^31^). This geometry also determines a different angle between the molecular axis and the chain direction (90° for PTO−Fe and 94.6° for PTO−Cu).

These same PTO−Fe coordination characteristics are encountered in the sparse filaments, where the main difference with respect to the monolayer is found in the adsorption configuration. Namely, the molecules engaged in PTO−Fe stripes are no longer lying parallel to the surface (Figure 7 b), with one of the peripheral phenyl rings being distorted away from the surface to give a tilted adsorption geometry. Notably, the calculated Fe adsorption height is higher in the sparse filament phase (2.22 Å) than in the compact phase (1.98 Å). The DFT calculations predict a significant electron migration to the organic layer, both from the Fe atoms and from the copper surface. In the case of the PTO−Fe monolayer, a Bader topological analysis produces an estimated −2.57 *e* charge transfer to the PTO molecules, very similar to what was calculated for the PTO−Cu structures. Moreover, the flat molecular geometry and the low adsorption height (ca. 2.2 Å) are similar to the PTO−Cu case, once more possibly leading to an overestimation of the predicted charge transfer in the Bader analysis. For the striped phase, however, the tilted adsorption configuration is expected to result in a more precise calculation of the Bader charges, and yields a charge transfer value close to an integer (1.99 *e*).

## Discussion

### Effect of charge transfer on the molecular structure and the adsorption configuration

The strong charge migration within the MOS, as highlighted by the ab initio models, is found to deeply influence both the molecular structure and the adsorption configuration of PTO molecules. With regard to the molecular structure, surface‐induced aromatic stabilisation is expected for acceptor molecules.^32^ This effect is particularly evident for the adsorbed PTO molecules, as the C=O bonds appear weakened and lengthened towards typical single C−O bond values (1.34 Å, compared to 1.23 Å calculated for gas‐phase PTO). Also, the shortening of the single C−C bonds bridging the two keto groups (1.43 Å, compared to 1.55 Å for gas‐phase PTO) provides further indication of a rather strong re‐aromatisation of the molecular board. These observations are relevant both to PTO−Cu and PTO−Fe MOS.

The large amount of net charge accumulated on the PTO molecules is likely to cause their flat adsorption configuration and their relatively small distance from the outer metal layer (ca. 2.3 Å). In fact, because of the polarisation response of the substrate (“image charge effect”^33^), molecular anions on a metallic surface generate a vertical, downward pointing electric dipole whose magnitude scales linearly with the molecular adsorption height. Reducing the vertical distance between PTO and the copper substrate is thus an efficient way of reducing the repulsion between equally oriented neighbouring molecular dipoles. The fact that PTO molecules arrange into extended 2D structures suggests that the residual repulsion energy cost is smaller than the binding energy resulting from the (attractive) van der Waals interactions and MO coordination, so that a compact assembly is energetically favoured.33c Moreover, a further electrostatic term is expected to additionally stabilise the Cu−PTO bonding: the positively charged metal adatoms carry an upward‐pointing electric dipole that partially screens the negative dipoles associated with the proximal PTO anions.9b Calculations on an isolated PTO molecule adsorbed on the Cu(111) surface are reported for comparison in the Supporting Information (Figure SI.3).

The DFT‐optimised structure of isolated PTO−Fe stripes shows an adsorption configuration different from that observed in the extended 2D MOS. The molecules no longer lie parallel to the surface but have one of the peripheral phenyl rings (ring B in Figure 7 b) pointing upwards, resulting in a tilted adsorption geometry (Figure 7 b, inset). This asymmetric configuration is ascribed to a different surface registry of rings A and B: ring A adsorbs with its extreme carbon atom (the 2‐carbon of the PTO molecule) on top of a surface atom and thus bends downwards to maximise its interaction with the substrate, whereas ring B is characterised by a less favourable surface registry and thus by a larger adsorption distance from the surface. The twisting of the aromatic board is also expected to be favoured by the significant charge transfer (1.99 *e* from Bader topological analysis), as previously observed for other aromatic species.14b, 32 These two effects (surface registry and higher conformational flexibility caused by charge transfer) are also present in the extended 2D MOS. However, PTO molecules are flat and have a small adsorption height within the 2D MOS because, as mentioned previously, this is an effective way of reducing the repulsion energy cost associated with neighbouring charged molecules^34^ which, in this case, is expected to be the most relevant effect. From the side view (Figure 7 b, inset), it also appears that the Fe atom is located farther away from the substrate than the C atoms within ring A of the PTO. Similar highly nonplanar MOS have been previously reported1a, 9b and were explained in terms of electrostatic screening (e.g., to attain the optimal magnitude of the surface dipole associated with the metal ion).

### Formation of the MO system and kinetics

The deposition of PTO on a clean Cu(111) surface was found to produce a metal–organic structure, with the ketone termini of neighbouring molecules bound to a Cu metal centre. The incorporation of adatoms is driven by the high electron affinity of PTO, which hosts four symmetric ketone groups. This geometry makes PTO an efficient polydentate ligand, with each pair of ketone groups providing two opposite chelating sites capable of inducing the formation of linear MO chains. Our DFT calculations suggest that the charge rearrangement leading to the formation of PTO−Cu MOS involves the donation of the 4 s electron from the Cu adatom to the molecule. The PTO−Cu−PTO geometry shown in the DFT relaxed structure of Figure 3 a is very similar to that formed upon chelation of Cu^I^ with bis‐phosphine monoxides,^35^ and the charge transfer predicted by our calculations is similar to an oxidation state I in 3D chemistry. In fact, Cu^I^ is stabilised by the interaction with weak acceptor ligand molecules, such as PTO, the electron affinity of which amounts to approximately 2.5 eV, whereas strong acceptors that favour a Cu^II^ oxidation state have electron affinities in the 4.0–5.5 eV range.^36^ Interestingly, annealing cycles of the PTO−Cu islands up to 450 K result in an Ostwald ripening process, indicating that the closed‐shell metal–organic interactions display a certain degree of reversibility at this temperature, allowing the formation of extended, defect‐free islands.

A significant change in the supramolecular arrangement was observed upon deposition of Fe atoms on the PTO−Cu islands. CO groups clearly preferentially coordinate to Fe centres rather than Cu ones since the Fe−O bond energy is roughly 40 % stronger than that of Cu−O, as previously reported for different complexes,^37^ for carboxylate‐based metal–organic coordination frameworks on metal surfaces10a, 18, 38 and tripyridyl ligands on Au(111).16a Our calculations confirm the higher Fe−O bond energy (ca. 1.7 eV vs. ca. 1.3 eV for Cu−O). This is likely caused by the additional interactions between the half‐filled d orbitals of Fe and the carbonyl lone pairs (Cu's 3d orbital is instead completely filled). This resulted in a different PTO−Fe−PTO coordination template, which, in contrast to the PTO−Cu−PTO structure, followed a perfectly square tetracoordinate geometry, as shown in the DFT relaxed structures of Figure 7, and very similar to the MO geometry in hemoglobin.^39^ Similarly to what was observed for the PTO−Cu complexes, DFT calculations also predict a metal‐to‐ligand charge transfer for PTO−Fe, with donation of one of the 4 *s* electrons of Fe to the molecule. As previously mentioned, this should contribute to the stabilisation of the metal–organic assembly through electrostatic screening.9b Finally, the lower brightness observed in STM for PTO−Cu complexes, in comparison to PTO−Fe ones, is most probably related to the slightly different adsorption height of the molecules in the two networks. In fact, when PTO is bonded to Fe, its core lies 0.03 Å and the two oxygens 0.3 Å higher than their positions in the PTO−Cu structures. Electronic effects are not expected to play any major role in this case, since the integrated density of states from 0 V to the imaging bias (−2 V) is 6.5 % higher for Cu−PTO than for Fe−PTO. As a consequence, if the electronic structure was to have any effect at all, it would cause the Cu−PTO structures to appear brighter than the Fe−PTO ones, that is, the opposite of what is experimentally observed.

Interestingly, our STM investigation revealed that, even after annealing to 550 K, the structure of the PTO−Fe MOS depends on the Fe deposition rate. In particular, compact islands formed by PTO−Fe chains were observed for low Fe deposition rates (Figure 5 and Figures SI.6 and SI.7 in the Supporting Information), whereas very thin and elongated assemblies of PTO−Fe chains (or even isolated chains) developed at high Fe deposition rates (Figure 6). Most probably, this difference is caused by a kinetic limitation of the rearrangement processes required for the formation of compact islands. STM images show the growth of Fe clusters at high Fe deposition rate (bright protrusions in Figure 6 a). These become preferred nucleation centres for the PTO−Fe chains, that grow radially out of them in an acicular phase with branches separated by 60° and oriented along the principal Cu(111) crystallographic directions (Figure 6). Once the strong MO coordination has occurred, the bond cannot be broken and it is unlikely that whole chains diffuse to form islands. Moreover, once formed, the chains act as barriers to molecular diffusion further hindering the development of compact assemblies. These mechanisms are expected to trap the system in a metastable phase, in contrast to the near‐equilibrium growth occurring in the low Fe flux case. Annealing temperatures of up to 550 K are not sufficient to promote the Fe−O bond breaking and the formation of 2D assemblies, consistent with the results of our DFT calculations (bond energy≈1.7 eV).

The formation of low‐dimensional supramolecular aggregates has been previously observed in the presence of repulsive interactions between charged elements.33b,33c, 34 Repulsive interactions must also be present in the systems reported herein, because of the charge state of coordinated PTO molecules (close to −2 *e*). However, we rule out any electrostatic explanation as the main reason for the 1D PTO−Fe stripes, because the nearest‐neighbour repulsive forces between anions carrying rather weak dipoles (ca. 5 D) is of the same order of magnitude as standard van der Waals interactions (e.g., on the scale of 0.01 eV). This is probably too weak to rationalise an interfilament separation (≫10 Å) that greatly exceeds the molecular size. Very simple equilibrium Monte Carlo models with low and high energy barriers for the breaking of the PTO−Fe bonds are able to qualitatively reproduce the two supramolecular assemblies observed at low and high Fe deposition rate, respectively (see the Supporting Information for details), substantiating the idea that the acicular structures in Figure 6 are kinetically driven.

Finally, we attempted to shed light on the low‐dimensional magnetic properties of the PTO−Fe MOS. We projected the spin‐resolved electronic ground state calculated for the MO chains onto Fe atomic orbitals. The spin magnetic moment was then obtained by integrating the spin density of the adatoms’ p and d states, from which the net Fe adatom spin polarisation was estimated to be approximately 1.87 μ_B_. This value is significantly lower than the same property likewise calculated for monoatomic Fe chains on Cu(111) (ca. 3.34 μ_B_ from ref. 40) and for trimesic and terephthalic acid−Fe chains on Cu(110) (ca. 3.4 and 3.3 μ_B_ from refs. 10a and 2b, respectively). We take this as an indication that the tetracoordination with carbonyl groups leads to a significant quenching of the iron spin magnetic moment, in turn suggesting that the structures investigated here are less likely to display low‐dimensional magnetic behaviour.

## Conclusion

Herein, we have described the preparation of a metal–organic assembly obtained by depositing PTO molecules on a Cu(111) surface, as well as the effects of codepositing Fe. Our combined experimental (STM) and theoretical (DFT) analyses allowed us to identify the interactions driving the formation of linear chelation complexes through symmetric metal–carbonyl coordination in the investigated MO structures. Our results reveal that polyaromatic molecules equipped with carbonyl groups can be successfully used to grow surface‐based MOS. In particular, our experiments reveal that PTO molecules deposited on clean Cu(111) terraces actively promote the capture of the metal atoms necessary to form large‐scale 2D PTO−Cu assemblies. The subsequent deposition of Fe atoms induces the disassembly of the Cu‐containing MOS, with the development of energetically favoured PTO−Fe complexes.

Our theoretical analysis reveals a sizeable charge transfer towards PTO molecules in both cases. As a result, PTO's LUMO is completely filled, with one electron coming from the adatom's 4 s levels and the other from the surface. The electrostatic interactions accompanying this charge transfer provide additional stability to the MOS, which remain intact when annealing up to 450 K. Annealing cycles can however still result in Ostwald ripening processes for the case of PTO−Cu coordination and in structural relaxation for the case of PTO−Fe at low Fe deposition rate, indicating that a degree of reversibility is allowed in these closed‐shell metal–organic assemblies. The different kinetic conditions associated with high Fe deposition rates produce sparse acicular structures consisting of individual or few connected PTO−Fe chain units that are stable up to 550 K. The formation of highly stable MOS suggests potentially interesting applications of these structures as functional organic coatings for organic electronics. In particular, the electrostatic layout of the MOS characterised by negatively charged PTO units partially balanced by metal linker cations generates a net dipole layer that could be used to tune the work function of a metallic electrode and thus to modify the charge injection barriers in organic‐based devices1a.

## Supporting information

As a service to our authors and readers, this journal provides supporting information supplied by the authors. Such materials are peer reviewed and may be re‐organized for online delivery, but are not copy‐edited or typeset. Technical support issues arising from supporting information (other than missing files) should be addressed to the authors.

Supplementary
