# Supplementary material for: Two‐Dimensional Ketone‐Driven Metal–Organic Coordination on Cu(111)
Source: Chemistry. 2016 Apr 13;22(24):8105–12. doi: 10.1002/chem.201600368 (PMC5074249; doi:10.1002/chem.201600368)
Supplement: Supplementary file 1 — Supplementary [file CHEM-22-8105-s001.pdf]

CHEMISTRY
A European Journal

Supporting Information

Two-Dimensional Ketone-Driven Metal–Organic Coordination on Cu(111)

Ada Della Pia,[a] Massimo Riello,[b] James Lawrence,[a] Daphne Stassen,[c] Tim S. Jones,[a] Davide Bonifazi,*[c, d] Alessandro De Vita,*[b] and Giovanni Costantini*[a]

chem_201600368_sm_miscellaneous_information.pdf

Supporting Information

Experimental methods

All STM experiments were performed in a commercial LT-STM chamber operated in ultra-high vacuum. The samples were scanned at 77 K with a chamber pressure of 5×10^{-11} mbar. The Cu(111) sample was prepared via multiple cycles of Ar^+ sputtering (1 keV) and annealing (up to 725 K) in a preparation chamber with a base pressure of 3×10^{-10} mbar. The PTO molecules were degassed at 373K for several hours prior to the initial deposition, with a shorter degassing (~10 minutes) at 423K before all subsequent depositions. The PTO was deposited onto the Cu(111) sample with an evaporation temperature of 423K. A typical PTO-Cu island formed after room temperature deposition is shown in Fig. SI.1. The bias voltage used during scanning was typically –2 V (occupied sample state imaging), with a tunnelling current of 2×10^{-11} A. The WSxM software[1] was used to process all STM images.

In all the experiments involving iron deposition, PTO was preliminary deposited onto the Cu(111). Two different Fe deposition rates were used: the low deposition rate was typically 8×10^{10} Fe atoms cm^{-2} s^{-1}; the high deposition rate was approximately 2×10^{11} Fe atoms cm^{-2} s^{-1}. It should be noted that enforcing precise rate values is difficult. The approximate values above were estimated by assessing the Fe atom coverage for a series of large-scale STM images relative to the deposition time and sample area.

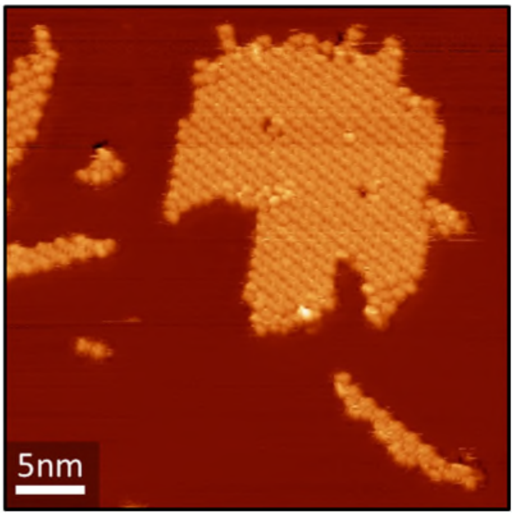

Figure SI.1. Small PTO-Cu islands that formed after depositing PTO on a room temperature Cu(111) sample without annealing.

Computational methods

Our DFT calculations and all postprocessing were carried out with the Quantum-ESPRESSO package suite[2]. Ultrasoft pseudopotentials[3] and PBE-GGA exchange-correlation[4] were used, the latter corrected by the vdW-DF functional[5] to account for dispersive interactions. The wavefunction energy cutoff was set to ~408 eV (30 Ry) for all simulations. The sampling of the Brillouin zone used a 4×4×1 Monkhorst-Pack grid, and a dipole correction was added to all metal slab calculations[6]. The Cu(111) surface was modelled by a three-layer slab, leaving ~12.5 Å of vacuum between periodic images. Structure optimisations were performed up to a force convergence threshold of 0.025 eV/Å (keeping the bottom layer constrained to the bulk). The simulated STM images were obtained by using the Tersoff-Hammann method[7].

Monte Carlo model

A lattice gas model was implemented in order to determine the effect of PTO-metal (M) bond strength on the morphology of the MOS. Near-equilibrium growth was simulated by using a standard equilibrium Monte Carlo sampling, with each particle in the simulation representing a PTO-M unit. These “structureless” particles were accommodated on a hexagonal lattice and allowed to interact through a two-component coupling, active between nearest neighbour

particles only. Namely, a higher attractive coupling was preset for bonding oriented along the three high symmetry directions to mimic the MO bonds, while a weaker attractive coupling was included to model secondary van der Waals interactions. In detail, the directional MO coordination bonds were represented by randomly assigning an integer "direction index" δ to each particle (δ =1,2,3 for the [011], [101] and [110] high symmetry directions, respectively). A single MO coordination bond was accounted for when two neighbouring particles had the same δ index, limited to two directional MO bonds per particle. The ratio between the MO coupling constant (J_{MO}) and the vdW one (J_K) was set to $J_{MO}/J_K = 50$, consistent with DFT bond energy estimates. Monte Carlo moves included standard particle swaps and rotations, the latter implemented by randomly changing the direction index δ. All configuration space samplings were carried out through the standard Metropolis algorithm.

Using this set-up to allow our "PTO-M like" particles to explore the available configuration space invariably produced low energy structures consisting of extended, ordered islands oriented along one of the available three directions (Fig. SI.2(a)). This is similar to the structures observed for PTO-Cu or PTO-Fe at low flux deposition (Figs. 2(a) and 5(a) of the main text). This 2D assembly pattern is also the expected thermodynamic equilibrium, since it maximises both the MO coordination (by maximising the coordination number through interchain links) and the van der Waals attraction (additionally pairing up neighbouring parallel MO chains).

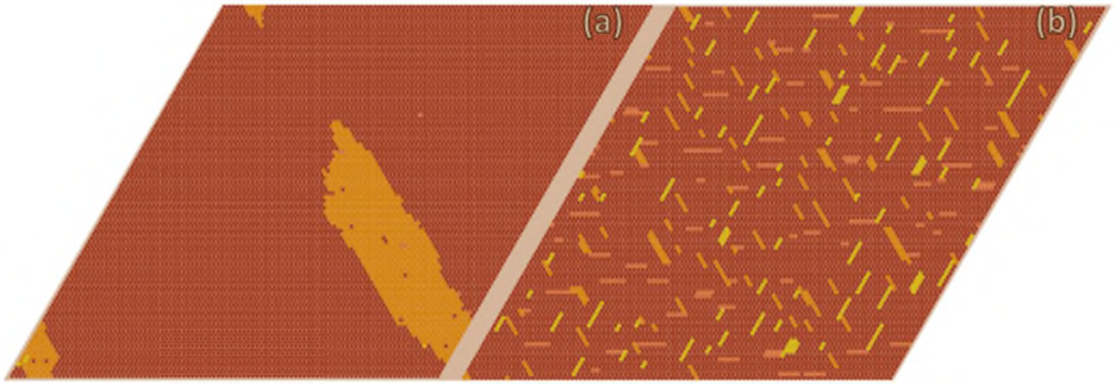

Figure SI.2. Equilibrium Monte Carlo simulations obtained including (a) no energy barrier and (b) a high energy barrier associated with PTO-M bond breaking/reforming events: large islands are produced in the former case, acicular structures in the latter. Three different colours (yellow, orange and red) have been used to indicate rows running along the three principal crystallographic directions.

The model predictions changed when the Monte Carlo sampling procedure was extended to include kinetic effects via the introduction a high energy barrier to the PTO-Me bond

breaking/reforming event (effectively boosting the MC moves that do not involve any coordination bond breaking, locally favouring initial aggregation over rebonding). In this case the coarsening of the PTO-Me like islands was hindered, leading to metastable supramolecular structures (Fig. SI.2(b)) that show a qualitative agreement with the acicular PTO-Fe phase (Fig. 6). These findings qualitatively suggest that the formation of low dimensional structures can be the result of quenching chemical reordering due to strong bond formation under high Fe deposition flux, while thermodynamic equilibrium would still be expected to involve the formation of mono-oriented PTO-M islands.

Synthesis

PTO was prepared according to the reported literature procedure[8].

Minority MO packing in PTO-copper assemblies

PTO molecules deposited on Cu(111) self-assembled by forming large islands with a rhombic unit cell (see figure 2 in the main paper). Rarely, a different molecular assembly with a different unit cell was observed growing from step edges, both on the upper and lower terraces (Fig. SI.8(a)). This structure involved pairs of Cu adatoms rather than single adatoms between neighbouring PTO molecules, in contrast to the majority PTO-Cu islands that formed on the terraces (Fig. 2(b)). The unit cell of this arrangement, shown in figure SI.8(c), had parameters **a** = 14.3 ± 0.4Å, **b** = 9.6 ± 0.5Å, θ = 43 ± 2°. The 2:1 adatom:molecule stoichiometry and the location of the islands at the steps may be linked. The local concentration of adatoms near the steps is in fact higher compared to the terraces, leading to a greater number of available atoms for MOS formation. At the edges of these islands, the adatoms involved in the MOS were observed to be mobile (compare Figs. SI.8(b) and (c)).

Substrate temperature dependence of the self-assembly

PTO depositions on both hot (423 K) and cold (143 K) Cu(111) were performed in order to examine the role of adatom concentration on the resulting assembly. As seen in figure SI.9(a), only the usual rhombic PTO-Cu assembly was observed when depositing on a hot substrate. The alternate packing was not observed, but no solid conclusions can be drawn

from this fact, as it may simply be that none were encountered due to their rarity. Depositing on Cu(111) held at 143 K had very different results as no MOS was formed (SI.10(b)). Small, bright aggregates of PTO were instead observed on the surface, most probably due to the reduced amount of Cu adatoms present at low temperatures, which thus hindered the formation of a MOS.

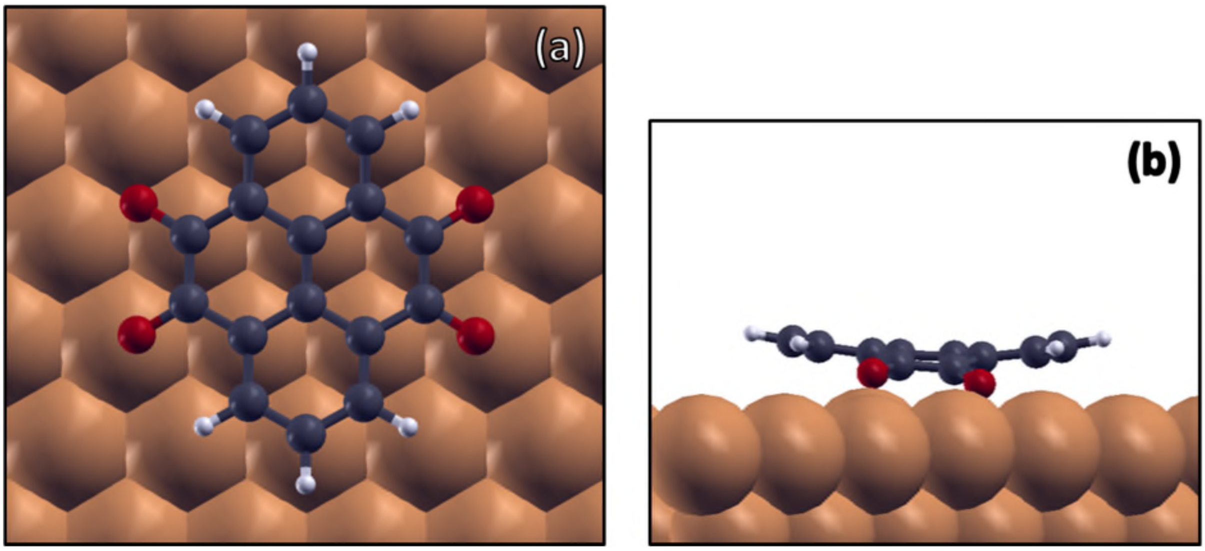

Figure SI.3. DFT structure of an isolated PTO molecule adsorbed on the surface (top and side view are reported in panel (a) and (b), respectively). In absence of adatoms, PTO molecules adopt a saddle-like configuration, with the oxygen atoms adsorbed at a closer distance from the surface than the carbon atoms of the aromatic core (1.97Å and 2.63Å, respectively). PTO molecules are expected to be in this adsorption geometry when deposited on the Cu(111) substrate held at 143 K.

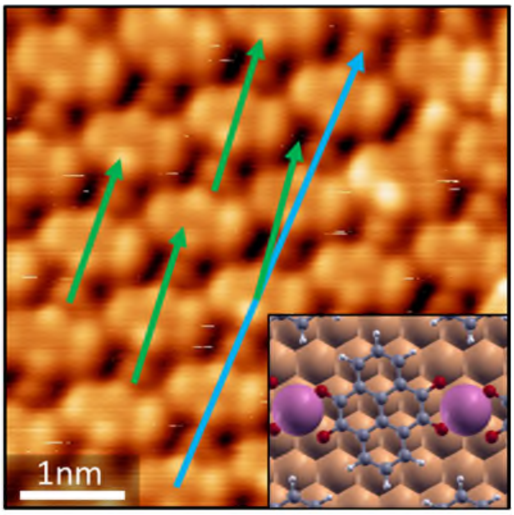

Figure SI.4. Close-up of PTO-Cu island showing the difference in orientation of the individual molecules (green) relative to the direction of the metal-organic chain (blue). The inset shows the DFT calculated adsorption configuration of a PTO molecule within a chain (compare with Fig. 2(a) of the main paper). The

molecule rotates by ~5° in order to bind to both Cu adatoms while staying close to the preferred adsorption site adopted by the isolated molecule (see Fig. SI.3).

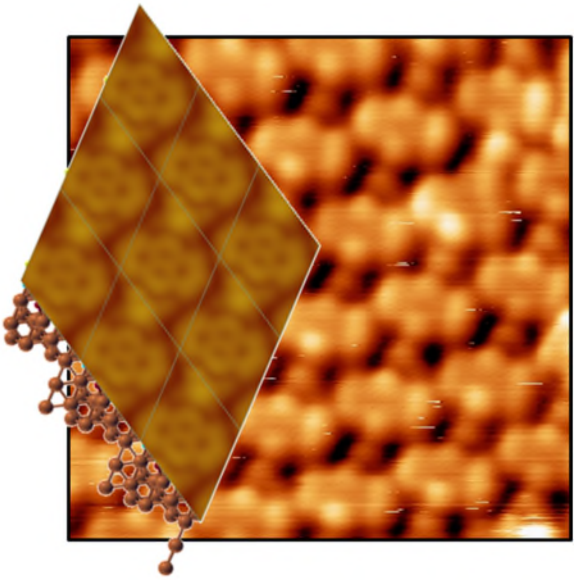

Figure SI.5. STM images of the majority PTO-Cu assembly and corresponding simulated STM image at -2 V obtained by using the Tersoff-Hammann approach[7]. Cu adatoms appear as protrusions, the PTO shape reflects the spatial distribution of the LUMO of a neutral PTO molecule in gas phase[9].

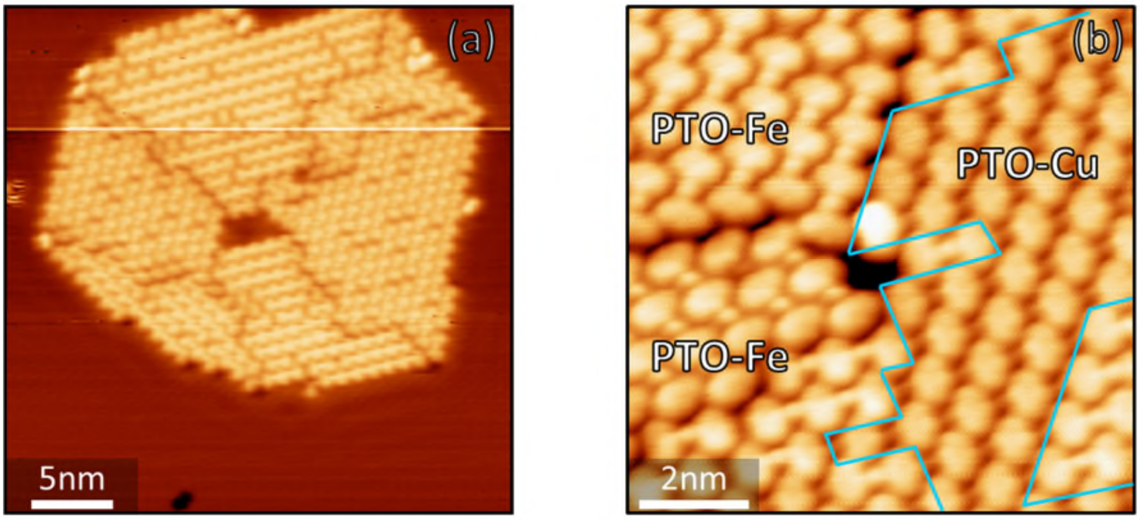

Figure SI.6. (a) Multi-domain islands are formed when depositing PTO and Fe atoms on a Cu(111) sample held at room temperature and annealing it to 450K. (b) When the Fe concentration is lower than a 1:1 PTO-Fe stoichiometry, areas including PTO-Cu chains are found in the multi-domain islands.

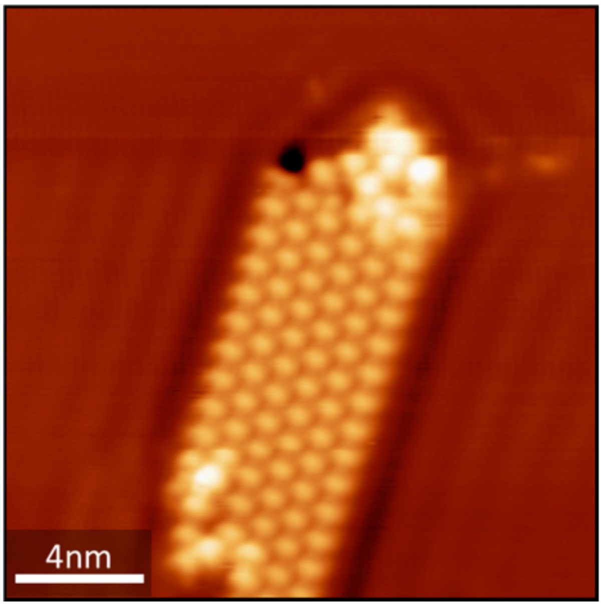

Figure SI.7. Example of a mono-domain PTO-Fe island formed after deposition of Fe onto pre-existing PTO-Cu assemblies, followed by annealing to 450 K. The Fe deposition was performed at a low rate (see main text) and at a coverage higher than what used in figures 3 and SI.5. Although present, the Fe adatoms are not easily recognised due to the low resolution of this image.

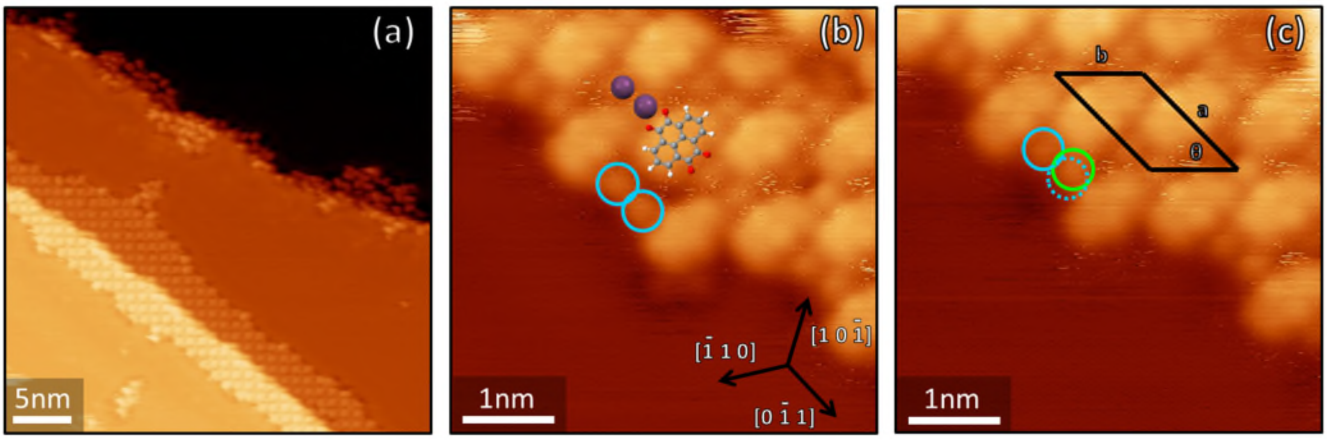

Figure SI.8. (a) Minority assembly formed by PTO molecules and copper dimers near the Cu(111) steps. (b) and (c): the mobility and position of copper adatoms at the island borders is shown via blue and green circles; the dotted blue circle in (c) indicates the previous position occupied by the mobile adatom relative to (b). A molecular model is also superimposed in (b), and the unit cell is shown in (c).

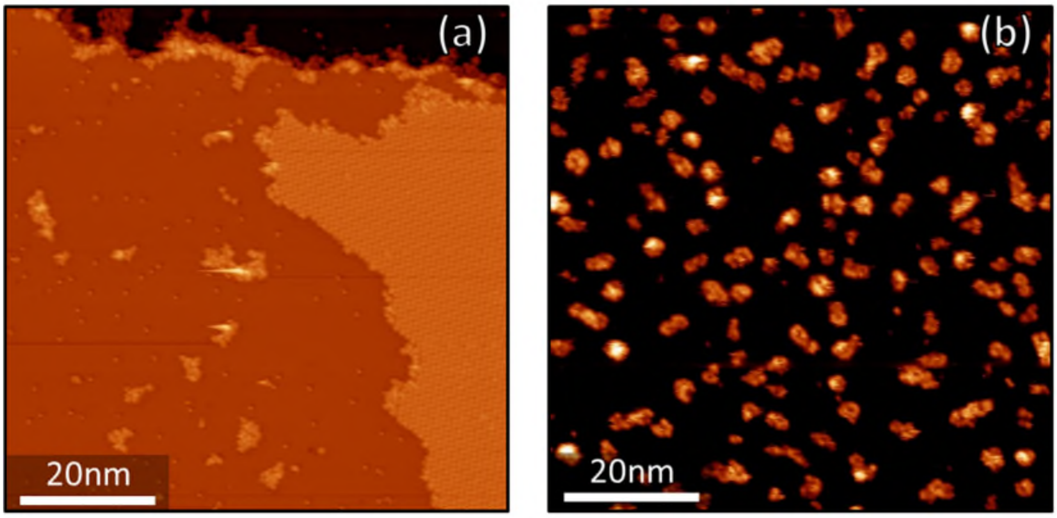

Figure SI.9. STM images acquired at 77 K after depositing PTO molecules on a Cu(111) substrate held at (a) 423 K and (b) 143 K.

References

[1] I. Horcas, R. Fernandez, J. M. Gomez-Rodriguez, J. Colchero, J. Gomez-Herrero, A. M. Baro, *Rev. Sci. Instrum.* **2007**, *78*, 013705-013701-013705-013708.

[2] P. Giannozzi, S. Baroni, N. Bonini, M. Calandra, R. Car, C. Cavazzoni, D. Ceresoli, G. L. Chiarotti, M. Cococcioni, I. Dabo, A. Dal Corso, S. de Gironcoli, S. Fabris, G. Fratesi, R. Gebauer, U. Gerstmann, C. Gougoussis, A. Kokalj, M. Lazzeri, L. Martin-Samos, N. Marzari, F. Mauri, R. Mazzarello, S. Paolini, A. Pasquarello, L. Paulatto, C. Sbraccia, S. Scandolo, G. Sclauzero, A. P. Seitsonen, A. Smogunov, P. Umari, R. M. Wentzcovitch, *J. Phys.: Condens. Matter.* **2009**, *21*, 395502-395501-395502-395519.

[3] D. Vanderbilt, *Phys. Rev. B* **1990**, *41*, 7892-7895.

[4] J. P. Perdew, K. Burke, M. Ernzerhof, *Phys. Rev. Lett.* **1996**, *77*, 3865-3868.

[5] M. Dion, H. Rydberg, E. Schroder, D. C. Langreth, B. I. Lundqvist, *Phys. Rev. Lett.* **2004**, *92*, 246401-246401-246401-246404.

[6] a) L. Bengtsson, *Phys. Rev. B* **1999**, *59*, 12301-12304; b) J. Neugebauer, M. Scheffler, *Phys. Rev. B* **1992**, *46*, 16067-16080.

[7] J. Tersoff, D. R. Hamann, *Phys. Rev. B* **1985**, *31*, 805-813.

[8] J. Hu, D. Zhang, F. W. Harris, *The Journal of Organic Chemistry* **2005**, *70*, 707-708.

[9] S. S. Naghavi, T. Gruhn, V. Alijani, G. H. Fecher, C. Felser, K. Medjanik, D. Kutnyakhov, S. A. Nepijko, G. Schoenhense, R. Rieger, M. Baumgarten, K. Mullen, *J. Mol. Spectrosc.* **2011**, *265*, 95-101.
